# Psychological factors are important to return to pre-injury sport activity after anterior cruciate ligament reconstruction: expect and motivate to satisfy

**DOI:** 10.1007/s00167-016-4294-8

**Published:** 2016-08-25

**Authors:** Sofi Sonesson, Joanna Kvist, Clare Ardern, Annika Österberg, Karin Grävare Silbernagel

**Affiliations:** 1 Division of Physiotherapy, Linköping University, 581 83 Linköping, Sweden; 2 School of Allied Health, Faculty of Health Sciences, La Trobe University, Melbourne, VIC Australia; 3 Aspetar Orthopaedic and Sports Medicine Hospital, Doha, Qatar; 4 Centre for Clinical Research Sörmland, Uppsala University, Eskilstuna, Sweden; 5 Department of Physical Therapy, University of Delaware, Newark, DE USA

**Keywords:** ACL, Return to sport, Patient-reported outcome, Patient perspective, Psychology, Activity level

## Abstract

**Purpose:**

To describe individuals’ expectations, motivation, and satisfaction before, during, and after rehabilitation for ACL reconstruction and to explore how these factors were associated with return to pre-injury sport activity at 1-year follow-up.

**Methods:**

Sixty-five individuals (34 males), median age 22 (15–45) years, scheduled for ACL reconstruction participated. Participants completed the International Knee Documentation Committee Subjective Knee Form (IKDC-SKF) and questions about expectations, satisfaction, and motivation pre-operatively and at 16 and 52 weeks after surgery.

**Results:**

Prior to surgery, 86 % of participants stated that their goal was to return to their pre-injury sport activity. Those who had returned to their pre-injury sport activity at 52 weeks were more motivated during rehabilitation to return to their pre-injury activity level, more satisfied with their activity level and knee function at 52 weeks, and scored significantly higher on the IKDC-SKF [median 92.0 (range 66.7–100.0)] at 52 weeks, compared to those who had not returned [median 77.6 (range 50.6–97.7)].

**Conclusion:**

Prior to ACL reconstruction, most participants expected to return to their pre-injury activity level. Higher motivation during rehabilitation was associated with returning to the pre-injury sport activity. The participants who had returned to their pre-injury sport activity were more satisfied with their activity level and knee function 1 year after the ACL reconstruction. Facilitating motivation might be important to support individuals in achieving their participation goals after ACL reconstruction.

**Level of evidence:**

Prospective cohort study, Level II.

## Introduction

Anterior cruciate ligament (ACL) injuries are common in young sports-active people (15–30 years). The injury leads to altered knee joint biomechanics and functional instability, and may require surgical treatment [9]. One of the most important reasons for performing ACL reconstruction surgery is to reduce knee instability in order to facilitate a return to the pre-injury physical activity level [8, 15, 18].

Patients have high expectations of recovery after ACL reconstruction; the majority expect good knee function and a return to the pre-injury level of sport [20]. However, these high expectations may not be fulfilled. Many athletes do not return to their pre-injury level of sports even though they are physically rehabilitated [19, 26, 27] and despite the fact that the goal of reconstruction and rehabilitation is to return to the pre-injury level [42]. Most patients (85–90 %) report good knee function after ACL reconstruction, but less than half return to their pre-injury competitive sport [7]. Unfulfilled expectations might also be associated with low satisfaction [13, 37].

Many factors influence whether an individual returns to sport [14, 18]. However, it is not fully understood what factors are associated with successful rehabilitation, resulting in a return to sports, and patient satisfaction after ACL reconstruction. Good physical function is a prerequisite to return to sport [1, 32, 40], and adequate post-operative rehabilitation is fundamental to facilitate recovery of the physical capacity required to participate in sport activity [15]. In addition, psychological factors and their impact on recovery, rehabilitation, and return to sport have received more attention in recent years [2, 5, 12, 14, 16, 18, 21, 28, 33, 34, 39]. Impaired mood has a negative impact on ACL rehabilitation [31], and in line with this, high optimism is associated with better knee function after rehabilitation [38]. High motivation also seems to be of importance for returning to sport after an ACL reconstruction [17, 21].

Rehabilitation is a long and demanding process that patients are often not fully mentally prepared for [23]. Data on patients’ expectations and motivation before an ACL reconstruction and during rehabilitation can provide enhanced knowledge of how these factors influence satisfaction and return to sport. Therefore, the purposes of the present study were to describe individuals’ expectations, motivation, and satisfaction before, during and after rehabilitation for ACL reconstruction and to explore how these factors were associated with return to pre-injury sport activity at 1-year follow-up. The hypotheses were that pre-operative expectations would be high and motivation would be important in order to return to pre-injury sport activity. Increased knowledge of these psychological factors can contribute to improve the current treatment protocols for this patient population.

## Materials and methods

For this prospective cohort study, all patients with an ACL injury, who were scheduled for an ACL reconstruction between January 2012 and June 2013 at one of three orthopaedic clinics in Sweden, were invited to participate. The inclusion criteria were: unilateral ACL injury; first time ACL reconstruction; and age 15–45 years at the time of ACL reconstruction. The exclusion criteria were: previous ACL reconstruction (to either knee); other major injuries to either knee (i.e. grade III collateral ligament injury, PCL injury, or grade III articular cartilage injury). Of 161 eligible patients, 65 patients completed the pre-operative questionnaire.

### Treatment

All patients received a hamstring graft for ACL reconstruction. Patients were treated according to standard Swedish clinical guidelines. According to the treatment algorithm, patients with ACL injury completed a period of structured rehabilitation (typically around 3 months) before a decision was made regarding surgery. Patients were advised to have ACL reconstruction if they continued to experience functional instability with recurrent giving way episodes or they wished to return to contact or pivoting sport or employment with high demands of knee function. Pre-operative information about the surgery, the healing process, the rehabilitation, and knee function after surgery should be provided to all patients before ACL reconstruction. The pre-operative information included rehabilitation time frames.

### Questionnaires

Electronic versions of a study-specific questionnaire (Table 1) and the Swedish International Knee Documentation Committee Subjective Knee Form (IKDC-SKF) [24] were sent to all participants via email before the scheduled ACL reconstruction, and at 16 and 52 weeks post-operatively (participants who did not have an email address were sent paper copies of the questionnaires). For each assessment, up to two reminders were sent. The assessments were timed to capture participants’ responses at clinically relevant times.

**Table 1:** The questions about participants’ expectations, motivation to return to pre-injury activity level, and satisfaction included in the study-specific questionnaire pre-operatively and at the 16-week and 52-week follow-up

Area	Question	Response model	Time point		
			Pre-operative	16 weeks	52 weeks
Expectations	Have you received information about the rehabilitation?	Yes/no/I don’t know	×		
	Do you feel prepared for the rehabilitation?	1–10; not prepared at all–very well prepared	×		
	When do you think you have completed the rehabilitation?	Numbers of months	×		
	When do you think you can run on even ground?	Numbers of months	×		
	When do you think you can jump on one leg?	Numbers of months	×		
	When do you think you can return to your earlier activity?	Numbers of months	×		
	Do you think that your current knee function matches the pre-op information?	1–10; function much worse than info–function much better than info		×	×
	What do you think is of importance for you to be able to reach your desired activity?	Choose the two most important factors: a good knee surgeon/a good physiotherapist/good compliance to rehabilitation/individual adjustments of the rehabilitation program/no unexpected incidents, e.g. disease/other	×	×	×
	Is your goal to return to your pre-injury activity and level?	Yes/no	×	×	×
Motivation	How important is it for you to return to your pre-injury activity level?	1–10; not important at all–very important	×	×	×
	Do you think it is possible to return to your pre-injury activity level?	1–10; not possible–possible	×	×	×
	How much are you willing to make the effort to return to your pre-injury activity level?	1–10; not willing at all–very willing	×	×	×
Satisfaction	Are you satisfied with your current activity level?	1–10; dissatisfied–very satisfied	×	×	×
	Are you satisfied with your current knee function?	1–10; dissatisfied–very satisfied	×	×	×
	If you were to spend the rest of your life with your knee function just the way it has been in the last week, would you feel: [11]	happy/satisfied/mostly satisfied/mixed feelings/mostly dissatisfied/dissatisfied/unhappy (1–7)	×	×	×

The study-specific questionnaire included questions about expectations, motivation to return to pre-injury activity level, and satisfaction (Table 1). The IKDC-SKF is a knee-specific questionnaire that evaluates symptoms, function, and sport activity. The instrument contains 10 items. The total score ranges 0–100 where 100 represents no limitations [24]. The IKDC-SKF has been translated and culturally adapted to Swedish (personal communication; manuscript under review).

### Ethical approval

Ethical approval was obtained from the Ethics Committee at Linköping University (Dnr 2011/450-31). All patients provided written informed consent prior to participation.

### Statistical analyses

Analyses were performed using the Statistical Package for the Social Sciences (SPSS) version 21 (IBM Corp, Armonk, New York, USA). Descriptive statistics were calculated for all outcomes. Mann–Whitney *U* tests and Pearson Chi-square tests were used to compare responses from participants who had returned to their pre-injury sport activity at the 52-week follow-up to those who had not returned. Significance was set at *P* < 0.05 for all analyses. Since this is a prospective exploratory study that for the first time describes individual’s expectation, motivation and satisfaction before, during and after ACL reconstruction, there were no known data to use for a power calculation. The post hoc evaluation revealed that the power of the study was 0.82 (one-tailed) for the question “Do you think it is possible to return to your pre-injury activity level?” at 52 weeks.

## Results

Questionnaires were sent to 161 patients before ACL reconstruction; 65 participants completed the pre-operative questionnaire at a median of 1 week pre-operatively and were included in the follow-up. Of these, 46 participants completed the 16-week questionnaire, and 43 participants completed the questionnaire at 52-week follow-up. The participants were a median 22 (range 15–45) years old at the time of ACL reconstruction. There were 34 males (52 %) and 31 females (48 %) (Table 2). Time from injury to surgery was a median 9 (0–128) months (Table 2).

**Table 2:** Participant characteristics and International Knee Documentation Committee Subjective Knee Form (IKDC-SKF) scores. Data displayed for the whole cohort and separately for those who at the 52-week follow-up had returned to their pre-injury sport activity, and for those who had not returned

Variable		All participants (*n* = 65)	Returned to pre-injury sport activity at 52 weeks (*n* = 17)	Not returned to pre-injury sport activity at 52 weeks (*n* = 25)
Age in years, median (range)		22 (15–45)	22 (15–41)	23 (16–44)
Sex, male/female		34/31	7/10	13/12
Time from injury to surgery in months, median (range)		9 (0–128)	8 (0–25)	11 (2–128)
Pre-injury sport activity, *n* (%)				
Football		31 (48)	9 (53)	12 (48)
Handball		8 (12)	3 (18)	2 (8)
Floorball		4 (6)	1 (6)	2 (8)
Enduro/motocross		3 (5)	1 (6)	0 (0)
Alpine skiing		2 (3)	1 (6)	1 (4)
Running		2 (3)	0 (0)	0 (0)
Gym training		2 (3)	0 (0)	2 (8)
Other		11 (17)	2 (12)	6 (24)
Pre-injury activity level, *n* (%)				
Elite		18 (28)	7 (41)	4 (16)
Competitive		36 (55)	6 (35)	17 (68)
Recreation		9 (14)	4 (24)	4 (16)
None		1 (2)	0 (0)	0 (0)
Goal to return to their pre-injury sport activity, yes/no		54/9	15/2	21/4
IKDC-SKF pre-operative, median (range)		51.8 (12.6–80.5)	58.0 (29.9–80.5)	48.3 (12.6–74.7)
IKDC-SKF 16 weeks, median (range)		61.5 (23.0–82.8)	54.6 (46.0–75.9)	61.5 (23.0–82.8)
IKDC-SKF 52 weeks, median (range)		83.7 (50.6–100.0)	92.0 (66.7–100.0)	77.6 (50.6–97.7)

### Pre-operative expectations

All except two participants reported that they received pre-operative information regarding surgery and rehabilitation. Most indicated that pre-operatively they felt prepared for rehabilitation (61 participants (95 %) estimated 6–10). Nine participants (14 %) estimated their rehabilitation would take 5–6 months to complete, 48 participants (76 %) estimated 7–12 months, and 6 participants (10 %) estimated more than 12 months rehabilitation duration. There were no differences between participants who had returned to their pre-injury sport activity at 52 weeks and those who had not returned, regarding the pre-operative estimated time to completion of rehabilitation, estimated time to be able to run/jump, or estimated time to return to pre-injury sport activity (n.s.).

At the 16- and 52-week follow-ups, participants estimated their current knee function slightly better compared to the pre-operative information about approximate time frames for achievement of rehabilitation goals (median 6, range 1–10 and median 7, range 3–10).

Participants stated that good compliance to rehabilitation, a good knee surgeon, and a good physiotherapist were the most important factors in order to be able to reach their desired sport activity (Table 3). There were no differences between participants who had returned to their pre-injury sport activity at 52 weeks compared to participants who had not returned regarding what factors they estimated were of importance (n.s.).

**Table 3:** Perceptions of what factors are of importance in order to be able to reach desired sport activity, pre-operatively and at follow-up. Participants chose the two most important factors

Factor	Number (%) who estimated that the factor was of importance in order to be able to reach their desired sport activity		
	Pre-operative	16 weeks	52 weeks
A good knee surgeon	38 (59.4)	16 (34.8)	13 (31.0)
A good physiotherapist	15 (23.4)	26 (56.5)	28 (66.7)
Good compliance to rehabilitation	58 (90.6)	43 (93.5)	37 (88.1)
Individual adjustments of the rehabilitation program	14 (21.9)	5 (10.9)	5 (11.9)
No unexpected incidents, e.g. disease	7 (10.9)	3 (6.5)	2 (4.8)
Other	1 (1.6)	1 (2.2)	2 (4.8)

Pre-operatively, 54 of 63 participants (86 %) stated that their goal was to return to their pre-injury sport activity, and at 16 weeks, 35 of 46 participants (76 %) had that same goal. Returning to the pre-injury sport activity at 52 weeks was not related to whether participants said their goal was to return at either the pre-operative or 16-week follow-up (n.s.). Satisfaction with knee function (1–7 scale; Table 1) at 52 weeks was not related to participants’ pre-operative goal for returning to sport activity (n.s.).

### Motivation

For most participants, it was very important to return to their pre-injury activity level (Table 4).

**Table 4:** Motivation to return to pre-injury activity level and satisfaction with current activity level and knee function. Data (median (range)) displayed for all participants (all) and separately for those who at the 52-week follow-up had returned to their pre-injury sport activity (returned), and for those who had not returned to their pre-injury sport activity

Question	Pre-operative				16 weeks				52 weeks			
	All	Returned	Not returned	*P* value	All	Returned	Not returned	*P* value	All	Returned	Not returned	*P* value
How important is it for you to return to your pre-injury activity level?^a, b^	10 (3–10)	10 (8–10)	10 (3–10)	n.s.	10 (1–10)	10 (8–10)	10 (1–10)	0.040	10 (2–10)	10 (9–10)	9 (2–10)	0.002
Do you think it is possible to return to your pre-injury activity level?^a, c^	9 (4–10)	10 (7–10)	9 (4–10)	0.019	10 (1–10)	10 (7–10)	8.5 (1–10)	0.054	9 (2–10)	10 (6–10)	8 (2–10)	0.011
How much are you willing to make the effort to return to your pre-injury activity level?^a, d^	10 (4–10)	10 (8–10)	10 (4–10)	n.s.	10 (1–10)	10 (8–10)	10 (1–10)	0.041	10 (2–10)	10 (7–10)	9 (2–10)	0.008
Are you satisfied with your current activity level?^a, e^	2 (1–10)	2 (1–10)	2 (1–4)	n.s.	4 (1–10)	3 (1–9)	5 (1–10)	n.s.	7.5 (1–10)	8 (5–10)	7 (1–10)	0.002
Are you satisfied with your current knee function?^a, e^	2 (1–8)	2 (1–8)	2 (1–5)	n.s.	6 (1–10)	6 (1–10)	5 (1–9)	n.s.	8 (1–10)	9 (6–10)	7 (1–10)	0.009
If you were to spend the rest of your life with your knee function just the way it has been in the last week, would you feel…^f^	6 (2–7)	5 (4–7)	6. (2–7)	n.s.	5 (2–7)	5.5 (2–7)	5 (2–7)	n.s.	3 (1–7)	2 (1–3)	3 (1–7)	<0.001

^a^Response model 1–10

^b^Not important at all–very important

^c^Not possible–possible

^d^Not willing at all–very willing

^e^Dissatisfied–very satisfied

^f^Response model: happy/satisfied/mostly satisfied/mixed feelings/mostly dissatisfied/dissatisfied/unhappy (1–7)

Participants who had returned to their pre-injury sport activity at 52 weeks, to a greater extent estimated pre-operatively that it was possible to return to their earlier activity level compared to those who had not returned at 52 weeks (*P* = 0.019, Table 4). Those who had returned to their pre-injury sport activity at 52 weeks were more motivated during rehabilitation to return to their earlier activity level compared to those who had not returned to their pre-injury sport activity (Table 4).

### Satisfaction

Satisfaction increased during rehabilitation. At 52 weeks, most participants were satisfied with their current activity level (median 7.5, range 1–10) and current knee function (median 8, range 1–10) (Table 4). Participants who had returned to their pre-injury sport activity at 52 weeks were more satisfied with their activity level (*P* = 0.002) and knee function (*P* = 0.009) at 52 weeks compared to those who had not returned (Table 4).

## Discussion

The main finding in the present study was that participants who had returned to their pre-injury sport activity at 1 year after surgery were more motivated during rehabilitation to return to their earlier activity level, compared to participants who had not returned to their pre-injury sport activity. Further, those who had returned to their pre-injury sport activity at 52 weeks were more satisfied with their current activity level and knee function. Prior to surgery, most participants stated that their goal was to return to their pre-injury sport activity and for the majority, this was of great importance. Consequently, motivation seems to be a key issue in ACL rehabilitation in order to reach a high activity level and satisfaction. Motivation is probably a prerequisite in order to achieve good adherence to the rehabilitation regime during recovery [10, 29, 35].

Since the standard practice is that the decision for ACL reconstruction is based on functional knee instability, the desire to return to contact or pivoting sport, or a knee demanding job, it is possible that a majority of patients receiving ACL reconstruction were highly motivated to undergo surgery and rehabilitation, and return to sport. Most participants in our study stated before their surgery that it was of great importance to return to their pre-injury activity level. Further, the majority of participants expected to do so. Patients’ expectations of recovery after ACL reconstruction are high [20], and expectations are a prognostic factor for physical recovery [30]. The current Swedish treatment model for ACL injury, including detailed pre-operative information, seems to provide adequate information to patients, supporting individuals to establish realistic expectations. In our study, participants’ estimations of time to accomplishment of rehabilitation goals and return to pre-injury sport activity were within the approximate time frames specified in the rehabilitation regime. Consequently, it is reasonable to suggest that participants had realistic expectations that reflected current published clinical guidelines and recommendations [1, 8, 22, 43].

Fulfilment of expectations is correlated with improved patient satisfaction [13, 37, 41]. In our study, satisfaction changed over time. Satisfaction with activity level and knee function was low before surgery and increased during rehabilitation. Returning to the pre-injury sport activity was associated with higher satisfaction. Consistent with this, recent research has also demonstrated that returning to physical activity participation is important for people’s satisfaction after treatment for ACL injury [3, 36]. This may be explained by the fact that return to sport is the goal for a majority of the individuals who undergo ACL reconstruction. Satisfaction following anterior cruciate ligament reconstruction is also strongly linked to patients’ perceptions of symptoms and function [25, 36]. Hence, patient satisfaction seems to reflect the outcome of the factors that are of greatest importance to the individual. Consequently, returning to the pre-injury sport activity seems to be of great importance for the individual and is associated with patient satisfaction.

Psychological factors have a critical impact on returning to physical activity following ACL reconstruction [2, 5, 12, 14, 16, 18, 21, 28, 34, 39]. We hypothesized that patients’ motivation during ACL rehabilitation would be essential for optimal outcomes in terms of returning to the pre-injury sport activity. In support of this, female football players who reported higher motivation to return to sports were more likely to return to playing football after ACL reconstruction [17].

Rehabilitation after ACL reconstruction has been acknowledged to be fundamental for the possibility to return to sport [15]. The rehabilitation process is long, and demands great effort from the individual. High motivation may result in good adherence to the rehabilitation protocol, and this might be important for reaching the levels of physical function that is recommended before return to sport [40]. In our study, participants who were more motivated to return to sport achieved that goal to a greater extent, which confirms other research showing a relationship between motivation and return to sport [6]. The clinical application of this finding is that facilitating motivation might be important to support individuals in achieving their participation goals after ACL reconstruction.

Motivation may need more attention in the clinical setting and may need to be addressed more specifically during rehabilitation. Current rehabilitations programs focus mainly on recovering physical function (impairment focus) and not on achieving psychological readiness to return to sport. Both physical readiness and psychological readiness to return to sport are important for returning, but they may not coincide [4]. A rehabilitation program may need to be complemented with a clearer psychological approach to maintain the patient’s motivation to return to the pre-injury sport activity. This may have the potential to improve returning to pre-injury sport activity and promote participation in physical activity. However, these hypotheses need to be tested in future research. Assessment of motivation before surgery has the potential to identify need for specific, individualized interventions aimed at improving psychological readiness to return to sport [12, 16]. Our study supports the need for further research to develop successful strategies to address psychological factors as part of rehabilitation after ACL reconstruction.

It is important to keep in mind that the participants underwent rehabilitation according to standard practice in Sweden and all except two participants received pre-operative information. It is likely that the provision of pre-operative information contributed to the fact that most participants felt prepared for rehabilitation and had realistic expectations. Consequently, accurate pre-operative information seems to be crucial to help patients establish realistic expectations. Individuals who have unrealistic expectations of ACL reconstruction may not be adequately prepared, and may need extra support during rehabilitation to maintain sufficient motivation. Although, since almost all participants in our study received pre-operative information, no comparison could be made with individuals who did not receive pre-operative information.

A strength of our study is that prospective data were collected before surgery, during, and after the rehabilitation period. The objective of the study design was to evaluate participants’ responses at clinically relevant times (i.e. immediately prior to the surgical intervention, at the time when sport-specific exercises are typically introduced, and when rehabilitation is expected to have been completed).

A limitation of the present study is the small sample size, and therefore, the results need to be confirmed in future studies. High motivation can be assumed to be a prerequisite to good adherence to a rehabilitation protocol; however, we did not empirically evaluate adherence in our study. Further, there are no data on whether every participant was cleared to return to sport or exactly when participants returned to sport. Instead, participants answered whether they had returned or not to their pre-injury sport activity at 52 weeks after surgery. This raises the potential for recall bias. However, given that returning to sport is a discrete and typically highly anticipated event, the chances of it being influenced by recall bias are likely to be low. Moreover, besides the psychological factors analysed in our study, other factors such as the surgery, rehabilitation and physical function may influence whether an individual returns to sport.

This study found that higher motivation during the rehabilitation was associated with returning to pre-injury sport activity. Facilitating motivation during the rehabilitation might have a beneficial effect on the person’s ability to achieve their participation goal.

## Conclusion

The majority of participants expected to return to their pre-injury activity level after ACL reconstruction. Motivation during rehabilitation was associated with returning to the pre-injury sport activity. Participants who had returned to their pre-injury sport activity were more satisfied with their activity level and knee function 1 year after ACL reconstruction compared to those who had not returned to their pre-injury sport activity.
